# How confidence intervals become confusion intervals

**DOI:** 10.1186/1471-2288-13-134

**Published:** 2013-10-31

**Authors:** James McCormack, Ben Vandermeer, G Michael Allan

**Affiliations:** 1 Faculty of Pharmaceutical Sciences, University of British Columbia, Vancouver BC, Canada; 2 Alberta Research Centre for Health Evidence, University of Alberta, Edmonton Alberta, Canada; 3 Evidence-Based Medicine, Department of Family Medicine, University of Alberta, Room 1706 College Plaza, 8215 - 112 Street NW, Edmonton AB, Canada

**Keywords:** Confidence intervals, Evidence based medicine, Statistical analysis, Statistical significance

## Abstract

**Background:**

Controversies are common in medicine. Some arise when the conclusions of research publications directly contradict each other, creating uncertainty for frontline clinicians.

**Discussion:**

In this paper, we review how researchers can look at very similar data yet have completely different conclusions based purely on an over-reliance of statistical significance and an unclear understanding of confidence intervals. The dogmatic adherence to statistical significant thresholds can lead authors to write dichotomized absolute conclusions while ignoring the broader interpretations of very consistent findings. We describe three examples of controversy around the potential benefit of a medication, a comparison between new medications, and a medication with a potential harm. The examples include the highest levels of evidence, both meta-analyses and randomized controlled trials. We will show how in each case the confidence intervals and point estimates were very similar. The only identifiable differences to account for the contrasting conclusions arise from the serendipitous finding of confidence intervals that either marginally cross or just fail to cross the line of statistical significance.

**Summary:**

These opposing conclusions are false disagreements that create unnecessary clinical uncertainty. We provide helpful recommendations in approaching conflicting conclusions when they are associated with remarkably similar results.

## Background

Most published reports of clinical studies begin with an abstract – likely the first and perhaps only thing many clinicians, the media and patients will read. Within that abstract, authors/investigators typically provide a brief summary of the results and a 1–2 sentence conclusion. At times, the conclusion of one study will be different, even diametrically opposed, to another despite the authors looking at similar data. In these cases, readers may assume that these individual authors somehow found dramatically different results. While these reported differences may be true some of the time, radically diverse conclusions and ensuing controversies may simply be due to tiny differences in confidence intervals combined with an over-reliance and misunderstanding of a “statistically significant difference.” Unfortunately, this misunderstanding can lead to therapeutic uncertainty for front-line clinicians when in fact the overall data on a particular issue is remarkably consistent.

A key concept of science is the formulation of hypotheses and the testing these hypotheses by observing a set of data. Typically in medicine one starts with an idea that a therapy will have an effect. A statistical test assumes that an intervention has no effect and this is called the null hypothesis. A statistical evaluation simply provides information as to how likely that the finding of a particular difference could be due to chance and if there really was no difference between the treatment groups.

We can NEVER prove a null hypothesis, meaning the intervention has absolutely no effect. However, we design clinical studies with the hope they will provide information to help decide if we should reject or fail to reject the null hypothesis. Rejecting the null hypothesis is often interpreted to mean the intervention has an effect; failing to reject the null hypothesis is interpreted to mean the intervention does not have an effect. These simplistic interpretations ignore important factors such as clinical importance, precision of the estimate, and statistical power.

A well-designed randomized controlled trial (RCT) is usually the least biased way to evaluate the difference between different therapeutic interventions. Unless an RCT has studied the entire population of interest, the observed difference or ratio that is found is called a point estimate because only a small sample of the entire population has been evaluated. A point estimate is typically presented with a 95% (or less commonly 99%) confidence interval (CI). A CI, while it has other interpretations, can be thought of as a range of numeric outcomes that we are reasonably confident includes the actual result.

The choice of a specific CI typically comes from the convention of a p-value of 0.05 representing a statistical significance. This threshold has been discussed as being arbitrary but has also been suggested to represent a reasonable threshold [1]. Any statistical threshold can be debated because a threshold depends on how comfortable one is that the results of a particular study may be due to chance. However, what cannot be debated is that this threshold was never developed to allow researchers or clinicians to make dichotomous conclusions that, if a p-value is greater than 0.05, the intervention has no effect and, if a p-value is less than 0.05, the intervention has an effect. When using CIs to assess statistical significance, the “no effect” cut-off occurs when the CI touches the line of 1 for relative risks or odds ratios, and 0 for absolute risks and weighted mean differences.

We have chosen three examples of this problem – a potential benefit of a medication, a comparison between new medications, and a medication with a potential harm. We will show this problem occurs with the highest-level evidence – randomized controlled trials and meta-analyses. We have framed these into three clinical questions:

1) In patients without a history of cardiovascular disease, do statins reduce mortality?

2) In patients with atrial fibrillation, when compared to warfarin, is apixaban more effective than dabigatran at reducing mortality?

3) In patients who smoke, does the use of varenicline increase the risk of serious cardiovascular adverse events?

### Statins

#### Example 1. In patients without a history of cardiovascular disease, do statins reduce mortality?

Statins are widely used in patients with and without established cardiovascular disease. An important clinically relevant question is: do statins have an effect on overall mortality in patients who have not experienced a cardiovascular event? Because of the relatively low baseline 5-year risk of mortality in this population (roughly 5% over 5 years), no single study has been powered sufficiently to provide a clear answer. For that reason, at least five different meta-analyses examining this question have been published [2-6].

The authors of these meta-analyses concluded the following:

•Studer *et al.*: “statins and n-3 fatty acids are the most favorable lipid-lowering interventions with reduced risks of overall and cardiac mortality.” [2]

•Thavendiranathan *et al.*: “statin therapy decreases the incidence of major coronary and cerebrovascular events and revascularizations but not coronary heart disease or overall mortality.” [3]

•Mills *et al.*: “We examined the impact of statin therapy on major events and found an important role in preventing all-cause mortality” [4] although this quote was not found in the abstract but rather the first line of the discussion.

•Brugts *et al.*: “statin use was associated with significantly improved survival.” [5]

•Ray *et al.*: “this literature-based meta-analysis did not find evidence for the benefit of statin therapy on all-cause mortality.” [6]

**Figure 1:**
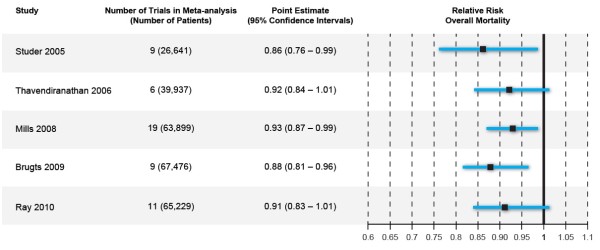
**Comparison of 5 meta-analyses examining relative risk of overall mortality with statin use in primary prevention.** Footnote: Brugts 2009 point estimate and confidence intervals are odds ratios (not relative risks).

Three groups of investigators felt they had found the pooled clinical trial evidence sufficient to state that statins reduce overall mortality; yet two others felt their evidence did not support statins reducing overall mortality. Figure 1 shows the characteristics and overall mortality relative risks of the five meta-analyses. Although the different meta-analyses included some different studies, overall the investigators used similar data and, not surprisingly, found similar results. The range of point estimates was 0.86 to 0.93 with an average point estimate of 0.90. The lower limits of the CIs ranged from 0.76 to 0.83 and the upper limits ranged from 0.96-1.01. The CIs overlap considerably and there is little meaningful difference in the results. The only “differences” lie in three meta-analyses [2,4,5] in which the upper limits of the CI fell just below 1.0 and just above 1.0 in the other two [3,6]. It appears the differing conclusions were due solely to a proclivity for a p-value of 0.05 and adherence to statistical significance as a dichotomous outcome.

### Novel oral anticoagulants

#### Example 2. In patients with atrial fibrillation, when compared to warfarin, is apixaban more effective than dabigatran at reducing mortality?

A new class of oral anticoagulants (OACs) has recently been released on the market. An important clinical question is which one of these new agents is the most effective; does either agent reduce mortality more than the “gold-standard” warfarin? Two separate studies compare two of the new OACs and warfarin [7,8].

Connolly *et al.* state “The mortality rate was 4.13% per year in the warfarin group, as compared with 3.75% per year with 110 mg of dabigatran (P = 0.13) and 3.64% per year with 150 mg of dabigatran (P = 0.051)” [7]. The authors do not make any specific conclusions on mortality differences between warfarin and dabigatran.

In contrast, Granger *et al.* (ARISTOTLE) concluded that “apixaban was superior to warfarin in preventing stroke or systemic embolism, caused less bleeding, and resulted in lower mortality” [8]. Interestingly, the press stated: “ARISTOTLE: A major win for apixaban in AF”, “the most positive yet” and “first of the three new oral anticoagulants to show a clearly significant reduction in all-cause mortality.” [9]

**Figure 2:**
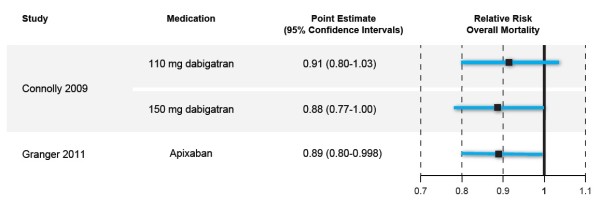
Comparison of 2 randomized controlled trials examining the relative risk of overall mortality with 2 novel oral anticoagulants versus warfarin in atrial fibrillation.

Figure 2 shows the relative risk for mortality with each drug (and dose) compared to warfarin. For all intent purposes the results are basically identical, particularly when comparing dabigatran 150 mg and apixaban.

The Granger paper [8] illustrates the importance that authors attach whether or not results cross the magical line of 1.0 and statistically significance. In Table Two of the article, the authors present 10 outcome results –for 9 they include the upper limit of the CIs with numbers to two decimal points. Yet for the mortality data, they show the upper limit number to 3 decimal points (0.80-0.998) [8]. Interestingly, for the mortality CIs in the abstract and the body of the paper, this 0.998 result was rounded down to 0.99 rather than the more correct rounding up to 1.00.

### Varenicline

#### Example 3. In patients who smoke, does the use of varenicline increase the risk of serious cardiovascular adverse events?

Varenicline is a new smoking cessation medication that is widely used. As with most new medications, the long-term or rare side effects are unknown. For that reason investigators have conducted meta-analyses of many small trials to try to identify any previously unknown adverse effects. Two meta-analyses have examined a possible risk of serious cardiovascular events with varenicline [10,11].

Singh *et al.* reported that “Our meta-analysis raises safety concerns about the potential for an increased risk of serious adverse cardiovascular events associated with the use of varenicline.” [10]

In contrast a later meta-analysis by Prochaska *et al.* reported that varenicline used for smoking cessation produced “no significant increase in cardiovascular serious adverse events associated with varenicline use” [11]. These authors go on to state “The consequence of inflated risk estimates, such as those from Singh and colleagues’ meta-analysis concerning the effect of varenicline on serious adverse events related to cardiovascular disease, can be unnecessary public alarm and real harm, since patients may discontinue their drug treatment out of fear of adverse effects and clinicians may recommend cessation treatments of reduced efficacy or discourage use of the drug treatment altogether.” [11]

**Figure 3:**
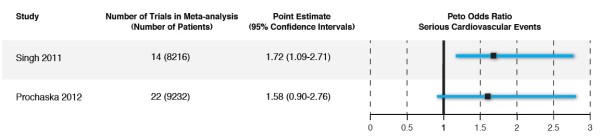
Comparison of 2 meta-analyses examining peto odds ratio of serious cardiovascular events with varenicline use in smoking cessation.

Figure 3 shows the characteristics and serious cardiovascular event peto odds ratios of the two meta-analyses. As with the previous examples, the two results are quite consistent with each other. The only apparent reason for the contradictory conclusions about risk was that the lower limit of the CI fell just below 1.0 in one meta-analysis, and was just above 1.0 in the other.

### Pragmatic interpretation of the included studies

Based on the evidence presented, we believe the following represents a reasonable and pragmatic interpretation of the results and how a clinician might use the information.

### Statins’ effect on overall mortality in primary prevention

If you were a betting person, you should bet that statins likely reduce mortality in primary prevention. The average point estimate in these meta-analyses was around 0.90 or a 10% relative reduction [2-6]. The relative reduction may vary from 0 to 20% but 10% is a reasonable approximation [2-6]. The baseline risk of mortality was approximately 5% [2-6], so using the 10% risk reduction gives a 0.5% absolute reduction. Therefore, we need to treat 200 primary prevention patients for one extra person to avoid death over 5 years. Conversely, 199 will not get a benefit in changing their risk of mortality. The CI also suggests the absolute reduction in risk could be as high as 1% or as little as 0%. Given the results we can confidently say it is very unlikely that statins increase mortality. Bottom-line, statins seem to have roughly a 1 in 200 effect on overall mortality in primary prevention.

### Novel anti-coagulants effect on overall mortality in atrial fibrillation

The evidence does not suggest any differences between apixaban and dabigatran and their effect on mortality compared to warfarin. As with the statins, dabigatran and apixaban likely do reduce mortality, approximately 10% with a CI of 0% to 20% [7,8]. The baseline death rate on warfarin was roughly 4% per year [7,8] so the reduction in risk would be 0.4% although it could be as high as 0.8% or low as 0%. Therefore, we need to treat 250 atrial fibrillation patients with apixaban or dabigatran instead of warfarin for one extra person to avoid death over 1 year. Conversely, 249 will not get a benefit in changing their risk of mortality. (Note: baseline death rates may vary considerably based on a specific CHADS score but we are using the average from the clinical trials included [7,8].) Based on the evidence, it is very unlikely that dabigatran or apixaban increase mortality compared to warfarin. Bottom-line, compared to warfarin, dabigatran and apixaban seem to have a 1 in 250 benefit on overall mortality in one year in atrial fibrillation patients.

### Varenicline effect on serious cardiovascular outcomes

If you were a betting person, you should bet that varenicline likely does increase the risk of cardiovascular events. The point estimate was roughly 60-70% but the effect could be as high as a 176% increase or an actual 10% reduction in cardiovascular events [10,11]. Importantly, baseline cardiovascular risk can vary dramatically. A 30-year-old female with no risk factors except smoking may have 0.5% 5-year risk or a 0.1% one year risk of a cardiovascular event. If varenincline increased risk by 60%, one extra cardiovascular event would occur for every 1667 patients treated. In patients immediately post-MI, whose CVD risk may approach 5% in the first year, varenicline may lead to one extra cardiovascular event for every 34 patients treated (7 of whom would quit smoking). In these patients, the benefits may still outweigh risks but a discussion around potential risks and options would be appropriate. Bottom-line, varenicline likely produces an increased risk of cardiovascular events but this risk may be <1/1000 for low risk patients to as much as in 1/34 for the highest risk patients. However, the benefits of smoking cessation are considerable.

## Discussion

It appears that medical authors feel the need to make black and white conclusions when their data almost never allows for such dichotomous statements. This is particularly true when comparing results to similar studies with largely overlapping CIs. Virtually all of the conclusion confusion discussed in this paper can be linked to slavish adherence to an arbitrary threshold for statistical significance. Even if the threshold is reasonable, it still cannot be used to make dichotomous conclusions.

Although we have selected three examples here, these are certainly not the only ones. In another example we considered, the authors of two meta-analyses of primary prevention with aspirin report the exact same point estimate and confidence interval 0.94 (0.88-1.00) but had differing conclusions [12,13]. We tried to select a small but representative group of examples that would be familiar to most readers.

We are not the first authors to write about the misinterpretation of CIs and statistical significance. About 60 years ago, RA Fischer introduced the p-value for hypothesis and significance testing [14]. Although 0.05 was suggested as a reasonable indicator for significance, he did assert the interpretation was open [14]. Over 30 years ago, a number of articles were published encouraging medical researchers to report their results with CIs [15-18]. CIs provide an estimation reflecting the potential range of effect rather than simply stating if results are statistically significant or not [15-17]. Unfortunately, this goal fell short as CIs are frequently used to define whether a result is or is not statistically significant. In the 60 plus year history, articles on application of statistical reporting continue to encourage authors to present their findings [17,19,20], allow readers to interpret results [14,17,19,20] and not use CIs strictly for reporting statistical significance [17,20].

We encourage authors to avoid statements like “X has no effect on mortality” as they are likely to be both untrue and misleading. This is especially true as results get “close” to being statistically significant. Results should speak for themselves. For that to happen, readers (clinicians and science reporters) need to understand the language of statistics and approach authors’ conclusions with a critical eye. We are not trying to say that the reader should not review the abstract but when authors’ conclusions differ from others, readers must examine and compare the actual results. In fact, all but one of the meta-analyses provided point estimates and CIs in the abstracts. This facilitates quick comparisons to other studies reported to be “completely different,” and to determine if the CIs demonstrate clinically important differences. The problem lies in the authors’ conclusions, which often have little to do with their results but rather what they want the results to show. We encourage journal editors to challenge authors’ conclusions, particularly when they argue they have found something unique or different than other researchers but the difference is based solely on tiny variations in CIs or p-value (statistically significant or not).

We are not suggesting the elimination of statistical testing or statistical significance, but rather that all people (authors, publishers, regulators etc.) who write about medical interventions use common sense and good judgment when presenting results that differ from others and not be so beholden to the “magical” statistical significance level of 0.05. We urge them to consider the degree to which the results of the “differing” study overlap with their own, the true difference in the point estimates and range of possible effects, where the preponderance of the effect lies and how clinicians might apply the evidence.

It appears that readers of the papers discussed here would be better served by reviewing the actual results than reading the authors’ conclusions. To do that, clinicians need to be able to interpret the meaning of CIs and statistical significance.

## Summary

Dogmatic adherence to statistical significance thresholds can lead authors to write dichotomized absolute conclusions while ignoring the broader interpretations of very consistent findings. These opposing conclusions are false disagreements that create unnecessary clinical uncertainty. Authors are encouraged to report data more pragmatically. Readers and clinicians are encouraged to compare the actual data and precision of the results rather than rely on the conclusions of the authors.

## Abbreviations

CI: Confidence intervals; RCT: Randomized controlled trial.

## Competing interests

We do not have any competing interest related to this article.

## Authors’ contributions

GMA conceived of the paper, collected the examples, completed the first draft of the figures, managed the manuscript, edited the article substantially, and is the guarantor. JM helped refine the idea, completed the first draft, edited the figures and edited the article. BV contributed to early discussions and crafting of the article and edited the article. All authors read and approved the final manuscript.

## Authors’ information

GMA is general practitioner and academic physician with focus in evidence-based medicine and knowledge translation. JM is doctor of pharmacy and an academic with a focus on evidence-based medicine and knowledge translation. BV is biostatistician and has written on statistical methodology and interpretation.

## Pre-publication history

The pre-publication history for this paper can be accessed here:

http://www.biomedcentral.com/1471-2288/13/134/prepub
